# A Review on New 3-D Printed Materials’ Geometries for Catalysis and Adsorption: Paradigms from Reforming Reactions and CO_2_ Capture

**DOI:** 10.3390/nano10112198

**Published:** 2020-11-04

**Authors:** Ahmad Soliman, Nahla AlAmoodi, Georgios N. Karanikolos, Charalabos C. Doumanidis, Kyriaki Polychronopoulou

**Affiliations:** 1 Mechanical Engineering Department, Khalifa University of Science and Technology, Abu Dhabi P.O. Box 127788, UAE; 100049412@ku.ac.ae; 2 Center for Catalysis and Separations, Khalifa University of Science and Technology, Abu Dhabi P.O. Box 127788, UAE; nahla.alamoodi@ku.ac.ae (N.A.); georgios.karanikolos@ku.ac.ae (G.N.K.); 3 Chemical Engineering Department, Khalifa University of Science and Technology, Abu Dhabi P.O. Box 127788, UAE; 4 College of Engineering and Computer Science, Vin University, Gia Lam, Hanoi, Vietnam; hdoumani@gmail.com

**Keywords:** additive manufacturing, 3D printing, catalysts, adsorbents, reforming, CO_2_ capture, carbon dioxide

## Abstract

“Bottom-up” additive manufacturing (AM) is the technology whereby a digitally designed structure is built layer-by-layer, i.e., differently than by traditional manufacturing techniques based on subtractive manufacturing. AM, as exemplified by 3D printing, has gained significant importance for scientists, among others, in the fields of catalysis and separation. Undoubtedly, it constitutes an enabling pathway by which new complex, promising and innovative structures can be built. According to recent studies, 3D printing technologies have been utilized in enhancing the heat, mass transfer, adsorption capacity and surface area in CO_2_ adsorption and separation applications and catalytic reactions. However, intense work is needed in the field to address further challenges in dealing with the materials and metrological features of the structures involved. Although few studies have been performed, the promise is there for future research to decrease carbon emissions and footprint. This review provides an overview on how AM is linked to the chemistry of catalysis and separation with particular emphasis on reforming reactions and carbon adsorption and how efficient it could be in enhancing their performance.

## 1. Introduction

According to Hannah Ritchie and Max Roser [1], the CO_2_ concentration in the atmosphere has presently exceeded 400 particles per million (ppm), which is the record for the last 800,000 years. It has also been reported that the concentration was about 300 ppm before 1900, and it increased sharply in the last century, with a global emissions total of 36 billion tons per year. Moreover, emissions increased at annual rates of 2.7% in 2018 and 0.6% in 2019, as compared to stabilized amounts between 2014 and 2017. In addition, Hertwich E. [2] reported an increase in the greenhouse gas (GHG) emissions of up to 11 giga tons, including a sharp peak of 120% between 1995 and 2015, as a result of the production of materials.

The increased societal need for alternative fuels and the necessity of the elimination of harmful CO_2_ emissions pushed the scientific community towards the development of a diversified portfolio of adsorbents and catalysts [3]. To add to that, scientific creativity along with the need for viable materials performing beyond their powder counterparts, led to novel architectures and robust structures.

In particular, exhaust gas treatment is nowadays mandatory in all sectors to secure a cleaner environment, free from harmful gas emissions, such as green house gases (GHG), e.g., carbon dioxide, and other gases, e.g., carbon monoxide, sulfur dioxide, etc., produced by anthropogenic activities [4,5,6]. Automotive transportation, growing by millions of cars/year, uses predominantly internal combustion engines burning fossil fuels to produce power and at the same time generates exhaust gases, primarily carbon dioxide (CO_2_), hydrocarbons (HC) and nitrogen oxides (NO). More than 65% of GHGs emitted by human activities are primarily CO_2_, with the majority coming from burning fossil fuels in automotive engines. Recent studies showed that 16% of the GHGs are methane emissions and 11% nitrous oxides [7]. Harmful emissions can severely affect the environment and enhance the greenhouse effect, upsetting the balance in many natural ecosystems. To resolve this issue, materials such as solid adsorbents and catalysts can be used to reduce the amounts of toxic pollutants such as CO_2_, NO_x_, H_2_S, etc. [8,9,10]. A large selection of zeolites, activated carbons, aluminas, and silicas has been reported and investigated in this context [11]. It has been found that some adsorbents behave more efficiently at low pressures and temperatures, while others exhibit better behavior at high pressure. Those differences would limit the selection of the CO_2_ capture process. Indicatively, recent studies [12] have shown a CO_2_ adsorption capacity of 9.51 wt.% using zeolites, while 360 mg/g CO_2_ has been reported using metal-organic frameworks (MOFs) [13]. Moreover, several researchers reported appreciable uptake capacities by using sol–gel-processed adsorbents [14,15] and hybrid adsorbent systems; approaches such as UV treatment were found to significantly enhance adsorbent performance [16].

At the same time, efforts toward producing renewable fuels with low carbon footprints (e.g., hydrogen) are being intensified. For the production of H_2_, different technologies can be employed, such as water electrolysis [17], water photo splitting and reforming [18]. Among available technologies, steam reforming (SR) is a process of hydrogen production with a long history of more than 80 years [19], whereby hydrocarbons are converted into mixtures of methane, hydrogen, carbon monoxide and carbon dioxide [20]. In addition, SR is also used to produce hydrogen from renewable and non-renewable feedstocks, such as methanol, diesel, jet fuels and liquified petroleum gas (LPG) [21].

There are many reports on using different feeds towards hydrogen production, such as naphtha [22], phenol [23] and ethanol [24]; and a plethora of catalytic systems, usually metal decorated oxides, including those using Rh as the metal [25], and Ceria-based [10] and MgO-based [26] as supports, have been reported. There is also significant research on non-noble metals, such as supported Fe catalysts [27], for hydrogen production. Such works achieved promising results in developing supported catalysts for applications that include SR and hydrogen production. Among the different hydrocarbon feedstocks available, methane has become more favorable due to its high hydrogen to carbon (H/C) ratio, producing more hydrogen and avoiding carbon deposition problems [19,28]. About 85% and 95% of world and US-based hydrogen production, respectively, are done using natural gas (CH_4_) SR [29,30]. Methane reforming includes two main reactions; firstly, the steam-methane chemical reaction (1), and secondly the water–gas shift reaction (2). At temperatures of 700–1000 °C and pressures of 3–25 bar, methane reacts via the catalyst to produce mainly carbon monoxide and hydrogen, and secondarily small amounts of carbon dioxide. Consequently, in the next step carbon monoxide reacts with steam towards forming carbon dioxide and hydrogen [19].

Steam-methane reforming reaction:

CH_4_ + H_2_O → CO + 3H_2_ (1)

Water–gas shift reaction:

CO + H_2_O → CO_2_ + H_2_ (2)

Wang, Y. et al. [31] reported the development of an Rh catalyst on a stable MgO–Al_2_O_3_ support to improve the volumetric efficiency of the methane SR process. The developed catalyst showed superior activity during short contact time and significant coke resistance at an equal ratio of carbon to steam for over 14 h time-on-stream. Yasuyuki M. et al. [32], investigated different support materials, including zirconia, alumina and silica, for nickel catalysts in the SR of methane where the effect of the chemical nature of the ceramic support was probed for SR at 500 °C.

Traditionally both categories of materials, i.e., CO_2_ solid adsorbents and reforming catalysts, are in powder form, but packed bed reactors are also used. The latter often suffer from disadvantages, including heat and mass transfer limitations, high pressure drops and uneven flows, hence giving rise to deviations in contact time and drop in selectivity. Catalytic materials are also typically used at high temperatures; thus, they suffer from mechanical and thermal stresses. The chemical fabrication methods usually employed for growth often give random porosity, size and shape of the catalyst/adsorbent. It is usually necessary to employ tedious experimental procedures, therefore, in order to achieve good control of the above material characteristics. This is where additive manufacturing (AM) can contribute. In an AM technique, a layer-by-layer addition of material takes place, while heat input secures fusion of the material, such that ideally, printing of a monolith can be achieved. Catalyst immobilization secured through 3D printing ensures fast recovery of the catalyst and continuous flow operation. AM has been used to make ceramic supports, such as SiO_2_, Al_2_O_3_, ZrO_2_ and SiC [33,34]; metal alloys, such as aluminum and steel [35]; and heterogeneous catalysts for several processes. The intrinsic characteristics of the catalysts are tuned through the fabrication method (e.g., catalytic performance, metal loading, shape and size). Various methods for catalyst preparation exist, including impregnation [36,37,38], co-precipitation [39,40,41], electrophoretic deposition [42,43], microwave [44], sol–gel [45] and vapor deposition-related techniques, such as chemical vapor deposition (CVD) [46] and atomic layer deposition (ALD) [47]. Wet chemistry procedures can be considered as mainstream but they suffer from certain drawbacks generally associated with low loading levels, non-controllable distribution of the catalytic species [42,43,48,49], sintering due to the limited catalyst immobilization and low mechanical strength.

In this review, we focus on AM fabrication of solid adsorbents for CO_2_ capture and metal supported catalysts for reforming of hydrocarbons, as reforming and CO_2_ capture constitute key industrial processes in the production of sustainable energy fuels and environmental remediation.

## 2. Additive Manufacturing Technologies

### 2.1. Introductory Remarks

AM (also known as 3D printing) is defined as a process of layer-by-layer successive addition of material(s) in order to build up three-dimensional objects. 3D printing is in juxtaposition to conventional subtracting processes such as machining, where raw material is being removed in order to obtain the final product structure (top–down approach). Moreover, 3D printing allows for convenient desktop fabrication by computer numerically controlled (CNC) machinery, using computer aided design (CAD) descriptions such as stereolithography (SLA) files via the printer controller to perform material deposition [50].

The first 3D printer was devised in 1981 [51], and for various reasons it was neither popular nor commercialized. Nowadays, 3D printers are gaining attention and adoption in many prototyping and manufacturing sectors, with a variety of printers exhibiting high accuracy, e.g., to 16 microns of layer thickness [52]. In addition, 3D printers can print composite structures with multiple component materials at the same time [53]. According to Wohlers [54], implementation of 3D printing keeps growing. Between 2015 and 2017, over one million desktop 3D printers were sold [55], with sales of industrial metal printers almost doubling in 2017 compared to the year before that.

3D printers are being developed rapidly, improving all features in order to minimize limitations. Clearly, not all printers have similar capabilities; for instance, 3D printers based on selective laser sintering (SLS) achieve high precision and powder material diversity at the expense of ownership and operation costs. Some ink-jet-based 3D printers can render fine detail rapidly, but for limited types of materials, which may be incompatible with, e.g., medical surgery [56,57,58]. In addition, 3D printers are generally limited by small working volumes, adding constraints to the size of printable parts. Moreover, 3D printers exhibit different energy requirements, with some of them using heat to melt a filament before droplet jetting, and others using lasers for solidifying liquid or melting solid materials, thereby necessitating different power levels. The most important limitations which printing technologies depend on are the feedstock materials, including thermoset photopolymers, thermoplastic powders and filaments, displaying different properties in their liquidization and solidification. Researchers reported the use of many different feedstock materials, such as polymers, ceramics, metals, biomaterials, live cells, etc. [59,60,61]. Modern engineering aims at producing large-scale, highly-efficient and versatile 3D printers to replace conventional manufacturing and construction, and therefore radically transform numerous industrial processes.

**Figure 1:**
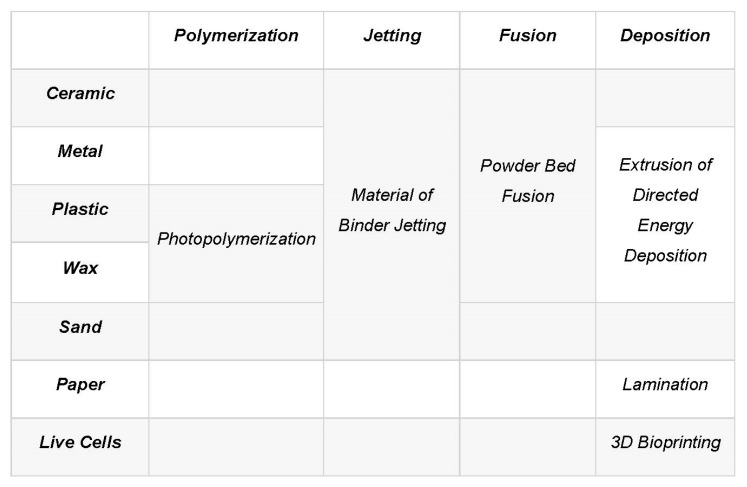
Infographic materials and 3D printing technology mapping [33,34,35,36,37,38,39,40,41,42,43,44,45,46,47,48,49,50,51,52,59,60,61,62,63,64,65,66,67].

The concept of layer-by-layer deposition enables printing of very complicated geometries, such as gyroidal structures inspired by nature [52], rapidly and with low cost, high resolution and good structural properties. 3D printing involves a wide range of operations and materials [33,52,59,60,62,63,64,65,66,67], based on which it is classified into several categories, as shown in Figure 1, according to the feedstock (material) used, liquidization and solidification methods used and type of energy used. A variety of techniques can be applied, such as robocasting, fused depositional material (FDM), selective laser sintering (SLS), ink jet printing (IJP), selective laser melting (SLM), etc. [68,69,70,71].

### 2.2. An Insight into Additive Manufacturing Techniques

AM includes several different 3D printing technologies, established and developed to process different classes of materials. Since each material has its unique properties, a dedicated treatment is needed in terms of liquidization conditions, shear thinning, mechanical and chemical properties of filaments and the suitable source and amount of energy. According to Parra-Cabrera et al. [72], these technologies are classified into many clusters: extrusion-based, stereolithography, inkjet printing, laminated object manufacturing (LOM), 3D welding techniques, powder-based technologies, etc. Each class represents one or more 3D printing technique for various materials; e.g., fused deposition modeling (FDM) is used mostly for polymers, and robocasting mainly for ceramics and metals. In catalysis, ceramics have gained special attention because of their use in high-temperature adsorption and separation, stressing the need for the requisite 3D printing techniques. Availability of ceramic material-based 3D printing technologies has indeed contributed to innovations in the catalysis research.

SLS and SLM can work with metals and ceramics in powder form; FDM uses the melting of polymers and extrusion of smooth pastes; robocasting or direct ink writing (DIW), similarly to FDM, deposits extrudates from a nozzle to form layers often from a ceramic slurry requiring sintering post-processing to achieve mechanical strength; the large variety of materials that can be 3D printed plays an important role on the AM. In this review, we will focus on 3D printing technologies to produce solid adsorbents and catalysts, and compare the resulting structures with conventional alternatives, e.g., powder beds [67,73].

#### 2.2.1. Robocasting

**Figure 2:**
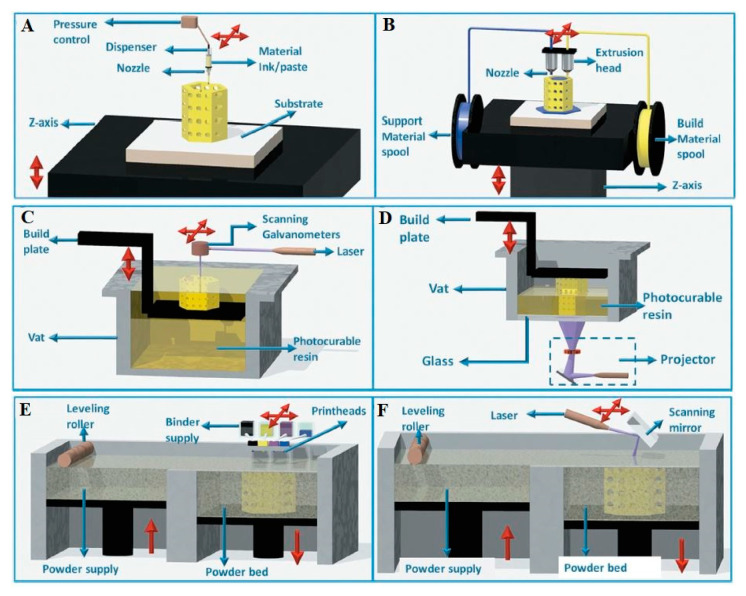
(**A**) Robocasting. (**B**) Fused deposition modeling. Stereolithography by (**C**) selective photo resin solidification is based on scanning a laser over the surface, or (**D**) by projecting an entire slice. Powder bed 3D printing methods by (**E**) 3D printing via binder deposition on a powder bed or (**F**) 3D printing via selective sintering on a powder bed. Reproduced with permission from [72]. Copyright The Royal Society of Chemistry, 2018.

The robocasting technique is a self-curable paste 3D printing technique used to fabricate ceramics, metals and composite slurries. It is known under several names in AM literature and practice, such as direct writing [74,75] and syringe extrusion printing [76]. Robocasting dispenses the ink/paste throughout a nozzle with certain printing conditions, such as extrudate viscosity and injection temperature. It usually uses ceramic slurry ink, with small metal-oxide loadings and metal-alloys for this printing type [77,78], by which the object is built by writing the required shape layer by layer. Figure 2A shows a schematic diagram of the 3D printer setup and component, where the ink from a syringe is extruded normally through a conical or cylindrical nozzle (inner diameter, d, typically ≥ 100 μm) by a computer-controlled robotic deposition system [79,80,81].

Robocasting leads to relatively thicker layers than stereolithography, and has been tested in building up catalysts, supports and substrates using ceramics and resins, metal oxides (e.g., Al_2_O_3_ and MgO [68]) and metal alloys, (e.g., AlSi_10_Mg, TiAl_6_V_4_, CoCr and Inconel 718 [69,70]). Robocasting is limited by the printing ink viscosity, which needs to be low enough to maintain its fluidity while being extruded out of the nozzle, but at the same time high enough to maintain the shape on the printed bead after extrusion [82].

#### 2.2.2. Fused Deposition Modeling

FDM is an AM technique that uses polymer filaments from a feed stock that goes through a heated nozzle to locally melt and get extruded to a thinner diameter to allow solidification during building, as shown in Figure 2B [75]. FDM is also known as fused filament fabrication (FFF). FDM belongs to the material extrusion family, as opposed to SLS and SLM, which use powder beds. The FDM is the most commonly used 3D printing technique due to its low-cost operation, its high resolution and the availability of thermoplastic polymers, which are the preferred source materials for this kind of printing [83]. Built volumes as large as 914 × 610 × 914 mm have been reported [84], and layer thicknesses as small as 20 microns [85] have also been achieved with some professional printers, along with speeds of up to 300 mm/s. Researchers have reported the usage of polymeric materials such as polylactic acid (PLA) (C_3_H_4_O_2_)_n_, acrylonitrile butadiene styrene (ABS) (C_8_H_8_)_x_·(C_4_H_6_)_y_·(C_3_H_3_N)_z_ [76,86,87] and polypropylene (PP) [88].

#### 2.2.3. Stereolithography

The stereolithography 3D printing technique is based on solidifying polymer resins using UV light, maintaining the selectivity of the materials. A photopolymer material is considered to be suitable for this family of techniques. The manufacturing process can be done either by laser scanning over resin surfaces (Figure 2C) or by projecting the entire slice (Figure 2D). Using computer-controlled UV heads, the UV light is directed to cross-sections upon their fabrication.

The stereolithography technique is limited to photosensitive materials such as polyethylene glycol diacrylate (PEGDA) hydrogel [89], which constitutes a challenge in catalysis industries. While a high-resolution output from the SLA technique is anticipated, coating the surface with active material after 3D printing can also play a significant role in the catalyst development. Some of these technologies are not directly related to 3D printing of ceramic materials for catalysis and adsorption, yet the polymeric materials which are 3D printed can be used as a matrix that can be coated with the ceramic material of interest.

#### 2.2.4. Selective Laser Sintering (SLS)/ Selective Laser Melting (SLM)

Selective laser sintering is a 3D printing technique using a powder bed and a laser source to sinter the polymeric or metal powder into solid structures according to a 3D design [90]. SLS is known by its productivity, although it is one of the most expensive methods, as, especially for the use of metals, it requires a long time but produces structures with high resolution compared to Robocasting. Notably, the resulting parts often have rough and grainy surfaces, which may need coating at the end to be smoothened [91]. According to the manufacturers [92,93,94], it is not preferable to reuse the unfused powder due to degradation by exposure to high temperature. SLS 3D printers use sintering to build up samples, whereas SLM printers use melting of powder beds [95].

SLS, also known as direct selective laser sintering and direct metal laser sintering [96], uses a powder bed, as shown in Figure 2A,E, and a laser source; the lenses and scanning mirror deflect the scanning beam to sinter the powder into the structure, as shown in Figure 2F [97]. The SLS is relatively expensive and has been used for limited types of materials [98].

SLM uses an arrangement similar to SLS, with different materials, e.g., mixed metal oxides. The technique involves complete melting of the powder, unlike SLS where sintering or partial melting takes place [99]. For almost all powder-based technologies, it is challenging to remove the unsintered/unmolten powder after the process is finished. SLM can play an important role in using 3D printing materials such as stainless steel [100,101], whereas SLS has been used in printing titanium alloys [102,103].

### 2.3. Factors Influencing the Quality of 3D Printed Structures

Undoubtedly, it is challenging to build a ceramic structure free of defects, especially for complex geometries. The need for ceramics is high in adsorption substrates because of their outstanding mechanical, thermal and chemical stability properties [104,105]. It is noteworthy that 3D printing of ceramics requires post treatment to remove the binder or unfused powder, potentially affecting or damaging the sample. Additionally, it can cause cracking and lead to defective parts with compromised mechanical properties of the final structure [106].

Many factors influence the 3D printing process and quality of the final product, with some being hardware-related [107]. Other parameters that play a vital role are:

**Layer thickness**: The layer thickness is the thinnest slice that can be printed, introducing a printer-specific limitation controlling final resolution and quality. As a rule of thumb, the smaller the layer thicknesses, the higher the quality. However, more time is needed to print the same structure compared to thicker layers.

**Material quality**: This is important for inks or powders (materials) used, for the resulting product to perform well after printing, i.e., by having favorable mechanical properties. These include, for instance, adequate yield strength, hardness, toughness and also chemical stability, avoiding rapid degradation with time and compromising the main function of these structures.

An indication of the material quality for a filament feedstock can include its diameter deviation. If the filament diameter varies over linear distance, this might cause inconsistencies in the thickness of a 3D printed wall, such as small bulges and cavities. This can result from an unstable flow of a 3D printer extrudate.

**Temperature:** Since 3D printers use a variety of types of materials, such as ceramics, polymers and metals, the processing temperature should be adjusted to ensure optimal results and a reliable printing process. For example, the acrylonitrile butadiene styrene (ABS) is extruded at a high temperature (240–250 °C) compared to PLA, which is cured at 200 °C [108]. For the case of the ABS the bed temperature should be kept at least 120 °C (given that T  >  T_G_), whereas for the PLA bed temperatures should be higher than 60 °C (applying the same criterion, T  >  T_G_), regardless of the bed material, for successful printing [109,110].

**Retraction:** This feature is similar to one used in CNC machines where the head is programmed to retract at safe distance from the object in order to avoid collisions with the structure, as for some complicated structures, such as fish net, the nozzle has to travel long distances managing the printing and the final quality. In this case, the best way to maintain good surface quality is to set a high speed for material retraction. This will prevent formation of small clods of material on the periphery.

**Inert Gas in Metal 3D Printing:** In the process of 3D printing metals, a controlled environment is a critical parameter. An inert gas (e.g., argon, nitrogen) environment is needed to minimize any impurities that would affect the final 3D printed structure [111,112]. The inert gasses are inactive and safe for the printing chamber, leading to high quality final products (3D printed structure). The inert atmosphere keeps away the risk of contamination from reactive gasses that exist in air, such as oxygen and carbon dioxide [113]. Techniques that use inert gasses include, SLS, Binder Jet (BJ), powder bed fusion and direct energy deposition.

### 2.4. Bringing AM to Catalytic and Adsorbent Structures

#### 2.4.1. Types of Catalysts/Adsorbents

The above-mentioned AM technologies can enable researchers in the catalysis field to print out various catalytic and adsorbent materials with complex architectures, potentially opening up new possibilities in gaseous flow dynamics; solid surface sorption and reaction; and heat/mass transfer in the substrate via diffusion and thermo/electric conduction. Important classes of materials include:

**Metal oxides**, which have important roles in catalytic reactions and separations, primarily as supports. They are typically powder-based materials, 3D printable via the aforementioned techniques. Metal oxides are typically used for catalyst substrates, undergoing another coating process with the active material. For example, Cu/Al_2_O_3_ was utilized by Tubio [114] for robocasting by dispersing Al_2_O_3_ powder into a Cu(NO_3_)_2_ solution. Arin et al. [115,116] utilized the ink jet technology in printing TiO_2_.

**Figure 3:**
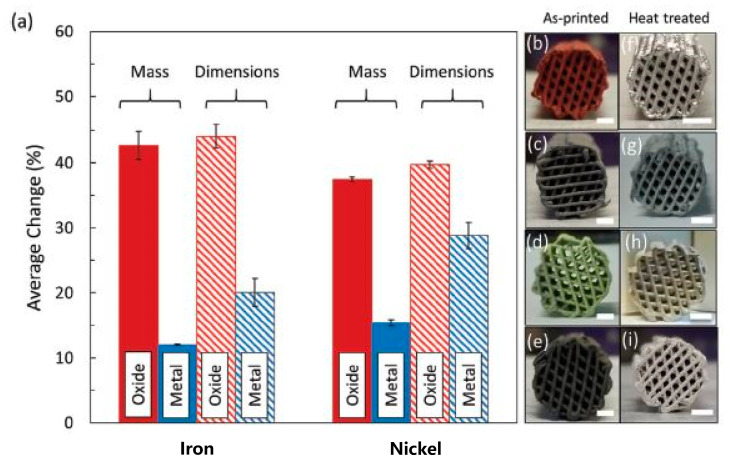
(**a**) Mass and linear dimensional changes for oxide and metal-derived structures made of iron and nickel. Due to the loss of oxygen after oxide reduction, oxide-derived samples have greater changes in mass and dimensions than their metal-derived counterparts [82]. (**b**,**f**) Samples derived from Fe_2_O_3_ particle-based inks. (**c**,**g**) Samples derived from Fe particle-based inks. (**d**,**h**) Samples derived from NiO particle-based inks. (**e**,**i**) Samples derived from Ni-particle-based inks. Scale bars at 2 mm. Reproduced with permission from [117]. Copyright Wiley-VCH, 2016.

**Metal Structures:** Shannon et al. [117] reported AM of a cellular metallic structure, using liquid ink, including metal oxide particles, to build the substrate, as shown in Figure 3a–i. The printed object went through a sintering process, affecting the structure linear dimension at its final stage of treatment. Results showed that the final structure exhibited high ductility and strength, with the capabilities of elastic and plastic energy absorption when the sample was projected to uniaxial load (4–31 MPa strength). The authors obtained an overall relative density of 32–49% with low stiffness (1–6 GPa) due to the architecture of the structure. They also found the shrinkage behavior of the oxide-derived structure to be superior compared to the metal-derived ones. Additionally, a more enhanced internal porosity has been observed for oxide-derived structures than for the metallic ones.

**Zeolite** structures have also been produced using extrusion-based technologies with FDM and robocasting techniques [65], where AM was employed for producing monoliths for CO_2_ removal. In addition, Couk et al. [118] prepared ZSM-15 zeolite using a three-dimensional fiber deposition technique. The structure, which is also composed of silica and bentonite, exhibits high potential for CO_2_/N_2_ and CO_2_/CH_4_ separation. The results showed enhanced mass transfer and pressure drop profiles while maintaining CO_2_ uptake and surface area.

It is worth noting that AM has also played a significant role in SR reaction. Kramer M. et al. [119] utilized the 3D printing technology to enhance the heat and mass transfer properties of 3D printed monolithic structures used as catalyst substrates made from ABS. This structure exhibited good thermal conductivity properties as well.

#### 2.4.2. Transferring Basic Concepts of Catalysis into 3D-Printing Technology

**3D printing’s effect on the metal particle size:** The metal particle size or the particle size of the active phase is critical, as it can increase the reaction rate for the so called structure sensitive reactions [120,121]. It is worthy to know that metal particle sizes can be tuned during the usage of additive manufacturing technologies in order to achieve various designs and structures. There are reported studies wherein researchers attempted to control, in different scale lengths, the metal particle size, i.e., from macroscale to nanoscale level. For example, a recent study done by Cheng Zhu et al. [122] reported on the fabrication of hierarchical nanoporous (hnp) Au metal particles by a combination of 3D printing and dealloying techniques. The latter dealloying step was used to create nanoscale porosity with pore sizes down to 10 nm. The hierarchy levels of structures and porosity are tuned by manipulating the 3D printing, alloying and dealloying conditions on the top of varying the ink composition. Replacing the microscale particles used in the ink with nanoscale particles would enable finer features and more detailed structures. Moreover, heat treatment (usually present in catalysis) often causes a volumetric shrinkage in the material which introduces a penalty to the mechanical properties/attrition of the final structure. In the case of hnp-Au metal particles, improved mass transport and reaction rates were reported.

**Metal-support strong interaction (SMSI) phenomena tuning through 3D-printing catalyst’s fabrication:** Undoubtfully, using 3D-printing fabrication methods for catalysts can tailor the metal-support interactions—a very important feature for every catalytic system governing the catalyst’s life span through hindering/facilitating metal sintering, metal reduction and other properties. Elkoro et al. [123] have reported on the 3D printed microstructured Au/TiO_2_ catalyst used for photo-catalytic production of H_2_. In the particular study, 3D printed titania (filamentous structure) was used as a support, decorated by Au nanoparticles (NP) either before or after the 3D-printing process. It was found that the Au addition step severely affects the catalyst activity, as it changes the distribution (homogenous vs. asymmetric) of the Au NPs for the cases of pre- and post-impregnation applied, respectively. The distribution of Au impacts the contact between Au NPs and support. This allows us to theorize that depending on the level of the fine control, different geometries of Au or another metal can be achieved, spanning from single atoms to small islands, or even clusters.

**Active site concentration:** Tailoring the population and the nature of the active site can affect not only the rate of the reaction of interest but also the type of catalytic chemistry that someone can expand into. In general, there are two ways to incorporate the active catalyst into the 3D-complex structure. This can be done either though physical mixing or through chemical binding.

**Figure 4:**
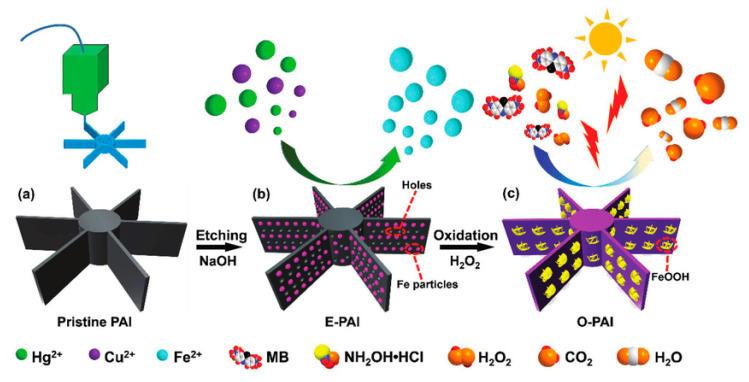
Schematic representation 3D printed AI preparation for water treatment. (**a**) Pristine PAI; (**b**) E-PAI (metal ions removal); (**c**) Fenton reaction catalyzer O-PAI [124,126]. Reproduced with permission from [124], Copyright Royal Society of Chemistry, 2013; [126], Copyright Wiley-VCH, 2018.

**(A) Physical mixing:** An example of physical mixing has been reported by Kitson et al. [124], who attempted to incorporate Pd/C catalyst and montmorillonite onto the walls of a 3D printed reactor using robocasting technique. The catalysts were subsequently used for Diels–Alder and amination reactions. No shape control or catalyst’s site accessibility was demonstrated in this report. The physical incorporation of the active catalyst has also been used along with the FDM technique. An example in this direction is reported by Skorski et al. [125] who added TiO_2_ into the ABS filament towards 3D printing of a composite structure which was used for the photocatalytic degradation of Rhodamine 6G. Another example comes from Sun et al. [126] who used an iron-containing PLA matrix to print structured impellers for the Fenton oxidation of aromatic compounds. To expose the Fe active sites and make them accessible to the reactants, base and H_2_O_2_ etching was applied. This is a way to tailor the active sites population on the 3D-printed structure (Figure 4).

**Figure 5:**
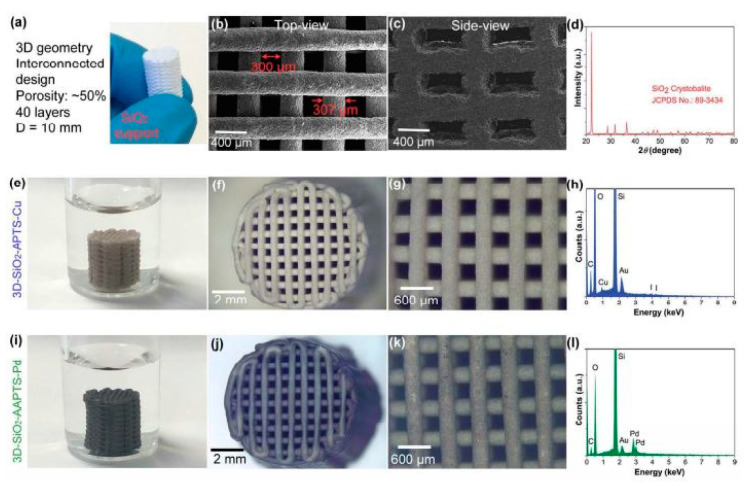
(**a**) SiO_2_ wood pile structure. SiO_2_ sintered structure scanning electron microscopy (SEM) images: (**b**) Top-view, (**c**) side-view and (**d**) X-ray diffraction XRD pattern. Optical images of the 3D-SiO_2_-APTS-Cu structure: (**e**) in the Kimble reactor, (**f**) top-view, (**g**) high-magnification top-view and (**h**) EDS spectrum. Optical images of 3D-SiO_2_-AAPTS-Pd structure: (**i**) in the Kimble reactor, (**j**) top-view, (**k**) high-magnification top-view and (**l**) EDS spectrum. Reproduced with permission from [128]. Copyright American Chemical Society, 2017.

**(A1) Physical Mixing after Surface modification:** This methodology includes post treatment of 3D printed structures. For example, Wang et al. fabricated an ABS 3D printed structure, which was subsequently functionalized by forming a coating of porous Cu-metal-organic frameworks on it through in situ growth process [127]. The range of the nanoparticles size on the 3D printed structure was 200–900 nm and it was used for methylene blue. In another work, Diaz-Marta et al. [128] demonstrated the addition of Cu and Pd active sites in 3D printed SiO_2_ monoliths after functionalizing the monoliths with amino silanes (Figure 5).

**(B) Chemical Binding:** In this methodology, the building blocks used in the printing contain the active sites, so there is no need for post-printing modification of the structure/surface. Towards this direction, manufacturing of stand-alone 3D photocatalytic titania geometries has been reported. To achieve this, in the titanium(IV)ethoxide precursor, the ethoxide ligands were exchanged with acrylic acid followed by SLA printing using a commercial 3D printer. Getting control of the molecular structures of the monomers gives the flexibility to manipulate the catalytic activity of the final geometries [129].

**Figure 6:**
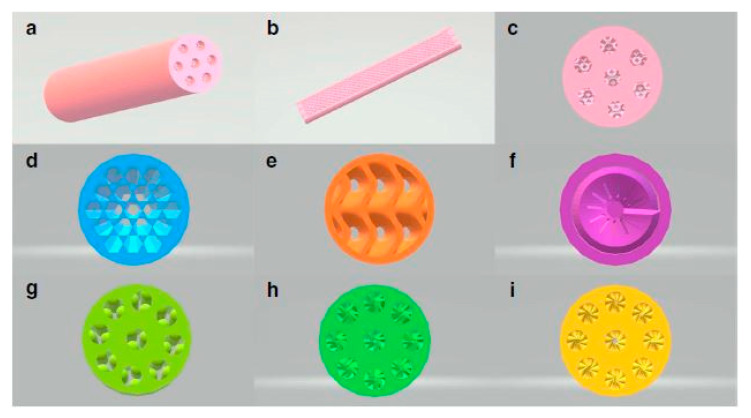
(**a**) Co-self-catalytic reactor (SCR)-1; (**b**) longitudinal section of Co-SCR-1; (**c**) cross-section of Co-SCR-1; (**d**) cross-section of Co-SCR-2; (**e**) cross-section of Co-SCR-3; (**f**) cross-section of Co-SCR-4; (**g**) cross-section of Co-SCR; (**h**) cross-section of Co-SCR-5; (**i**) cross-section of Co-SCR-6. Reproduced with permission from [130]. Copyright Nature, 2020.

**Figure 7:**
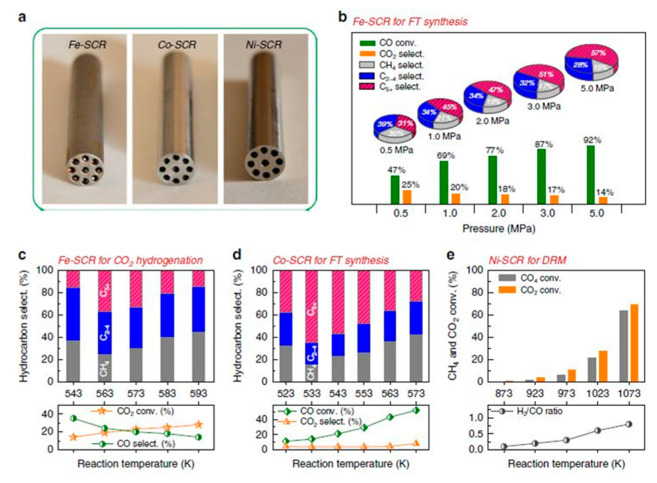
(**a**) The physical SCRs after polishing the outer surface. (**b**) Fe-SCR for F–T synthesis. (**c**) Fe-SCR for CO_2_ hydrogenation. (**d**) Co-SCR for F–T synthesis. (**e**), Ni-SCR for CO_2_/CH_4_ (dry reforming). Reproduced with permission from [130]. Copyright Nature, 2020.

**Deactivation:** Deactivation of the catalytic systems is a crucial factor governing not only the process efficiency in terms of catalytic activity and selectivity, but also the potential of commercialization of a catalytic system. There are different phenomena contributing to deactivation, including metal sintering (due to excessive heat), chemical poisoning and fouling. In the work reported by Qinhong Wei et al. [130] the concept of the self-catalytic reactors (SCRs) was proposed towards enhancement of the Fischer–Tropsch (F–T) reaction catalytic activity and the production of liquid fuel/syn-gas. This study also reported that by adopting a different geometry (Figure 6), a tuning in the selectivity is reached (lighter vs. heavier hydrocarbons). For a better understanding of this effect, someone should consider geometrical parameters, such as inner surface and channel volume, while calculating the liner velocity (V_linear_) and passage time (T_passage_) of the feed gas (here syngas). It is worth defining the T_passage_ parameter—the time required for syngas to cross the channel (T_passage_ = V_channel_ / F_CO+H2_) (Figure 7). Additionally, it has been proposed that with the right channel geometry, the local overheating can be avoided—a rather regular problem in the traditional F–T catalysts. Another important structural aspect that is introduced by the 3D-printed catalysts is the fine tuning of the contact times and fast purging of the desired reaction products, thereby avoiding further catalytic conversion which could potentially lead to side-products (precursors of coking). This can potentially lead to another strategy towards controlling coking over this type of reactions. In addition, outstanding heat management in the F–T reaction has been reported by Fratalocchi et al. [131] in the case of Co/Pt/Al2O3 catalyst packed into the periodic open cellular structures (POCS), the latter being 3D-printed.

### 2.5. Metrology

Despite the benefits of 3D printing in producing prototypes with controlled geometrical features, there are still challenges associated with the measurement and characterization of the final 3D printed product. Quality control is essential to prove and reach the desired repeatability, reproducibility, reliability and precision [132]. In order to control the quality of the final products, researchers have investigated the above factors and tried to standardize the characterization methods through ASTM and ISO standards [133].

The number of publications on powders for AM has recently increased. Slotwinski et al. [134] tried to define the technical challenges on characterization of metal 3D printed powders. Recent efforts led to release of ASTM F3049 standard guide for characterizing metal powders used for AM processes. Clayton [135] reported two types of powders with the same measured shear behavior to have different dynamic behavior. Additionally, Lyckfeldt [136] reported that steel powders of the same particle size distribution can exhibit different flow properties, which can affect the process performance and thus the final object. Work by Starr et al. [137] and Murr et al. [138] has been carried out in similar sets of experiments using gas-atomized steel.

Slotwinski et al. [134] summarized some key technical needs for AM processes. Regarding material properties, these include high-quality 3D printed parts for high-stress operation; and better understanding of the relationship between powder properties and part properties. In addition, there are only a few commercially available powders usable for 3D printing. Thermal process phenomena, such as quenching, heating and cooling, take place in-situ during 3D printing, making it difficult to measure and understand the effects to the process. Additionally, there are no standardized methods for certifying the 3D printing process’s input materials. The surface finish and dimensional accuracy of 3D printed metallic objects are generally poorer than those from conventional material manufacturing and removal processes. Additionally, their size and production time are limited and lacking with respect to certain production standards, while there are only two formats for general CAD files in use STL and AMF (stands for additive manufacturing file). Although AM overcomes some STL limitations, it is still not compatible with all systems, and is not widely used.

Various efforts for optimizing the printing set up and manufacturing process quality are being carried out to enhance the final product output. For instance, Edward et al. [139] developed a self-heated bed for fusing polymeric, ceramic or metallic powders used in AM technology to reduce the residual stresses and minimize the amount of wasted energy, leading to more stable material structures. Further developing AM methods and minimizing the complexity of 3D printing technologies are still on going, intending to make usage more reliable and dependable.

Townsend et al. [133] discussed the surface texture metrology for 3D printed metal parts and identified many surface measurement technologies and strategies based on both material nature and structure topography. In general, mechanical properties are heavily influenced by topography and density. Firstly, contact-based probe profilometry methods are suggested for robust surfaces, unlike highly porous surfaces, in which structural damage could ensue instantaneously upon touching. Moreover, the probe tip diameter and conic structure can be carefully resized for different application sizes and feature dimensions.

On the other hand, non-contact techniques, for instance, electron microscopy using energetic electron beams, should be considered, as they do not affect the properties of the printed materials. A variety of measurement technologies are utilized in surface characterization, such as contact stylus [140,141,142,143], confocal microscopy [144,145], focus variation microscopy [146], coherence scanning interferometry [147], chromatic confocal microscopy [148], conoscopic holography [140], atomic force microscopy (AFM) [143], elastomeric sensor [149,150], optical microscopy [143,151], SEM [96,144], X-ray computed tomography [152,153] and Raman spectrometry [141]. Researchers mainly use the probe-contact based method to measure the profile texture. Some pre-tested textures are also supported by well- established standards from ISO and ASME, such as ISO 3274, ISO 4287, ISO 4288 and ASME B46.1. On the other hand, 2D area topography for metal characterization is highly dependent on optical measurement devices, such as confocal microscopy and focus variation microscopy; most research has utilized SEM for 2D imaging along with optical microscopy. Although X-ray computed (XRC) tomography is not widely used because of resolution, speed and cost limitations, it can perform 2D area analysis by extracting information from 3D volumetric data, with no limitation on the complexity of the structure geometry.

**Figure 8:**
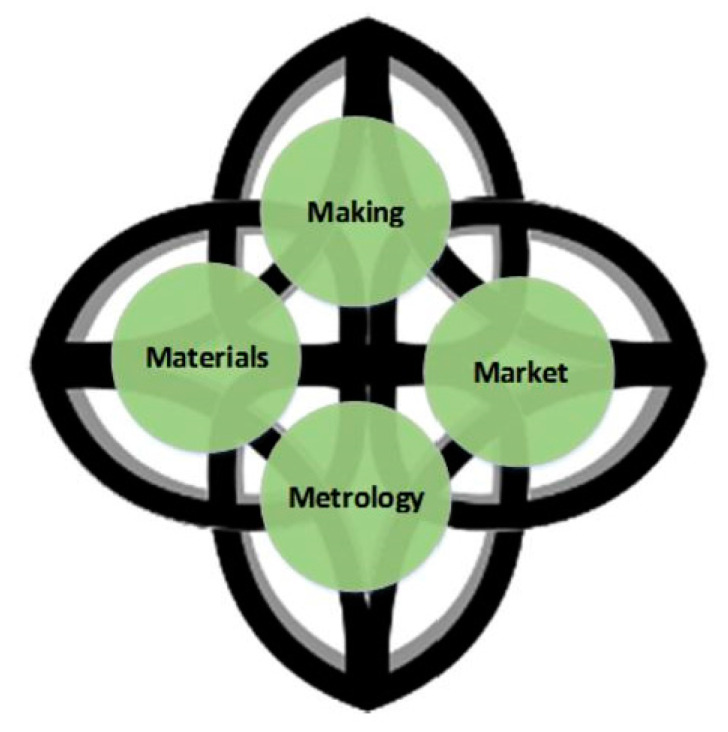
The four Ms (4Ms) of AM: materials, making, metrology and market. Reproduced with permission from [154]. Copyright Elsevier, 2017.

According to Syed A.M. et al. [154], to ensure successful performance of the final 3D printed product before it goes to the market, the product needs to meet certain standards and achieve the desired specifications when testing an appropriate metrological toolset. The inter-twinned relation between the four Ms, i.e., material, market, metrology and making, are described in Figure 8.

Unceasing challenges for 3D printing technologies lie in developing a user-friendly system to control deposition speed, location, resolution and dimensional accuracy, so as to optimize the impacts of manufacturing process conditions on final part quality. AM for metals requires a shielding atmosphere to prevent sample oxidation and ensure high quality output. It also requires a highly energetic source of thermal energy for melting fusible metals, such as a laser; in polymers, low-powered heat sources are sufficient to complete the process, which can be implemented by light or ultrasonic waves. Ceramics also require low energetic thermal sources, since they are poor thermal conductors, while power can account for undesirable thermal shocks during the process.

**Figure 9:**
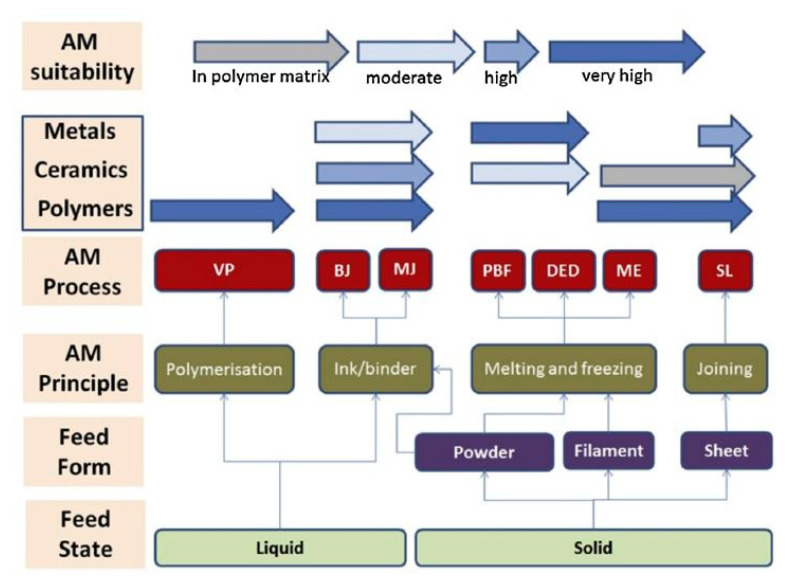
Schematic of the relative suitability of AM for three major types of materials (polymers, ceramics and metals) in various feed forms and states using ASTM processes: binder jetting (BJ); directed energy deposition (DED); material extrusion (ME); (4) material jetting (MJ); powder bed fusion (PBF); sheet lamination (SL); and vat photopolymerization (VP). Reproduced with permission from [154]. Copyright Elsevier, 2017.

**Figure 10:**
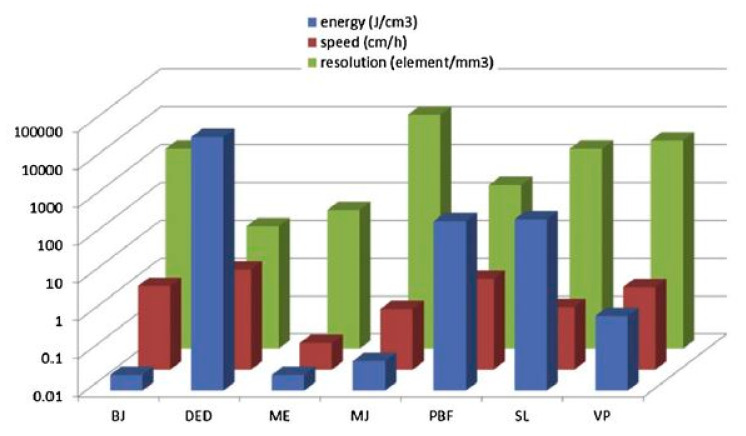
Energy used, speed and resolution of fabrication in different AM techniques. Reproduced with permission from [154]. Copyright Elsevier, 2017.

It is worth noting the importance of material considerations; e.g., a printer designed to print alloys may not be able to print composites, or even single elemental materials. AM materials should be selected in conjunction with the respective printing technology, heat, fusibility and depositional properties. While AM can address scalability issues, it is more important to realize that there is no one-size-fits-all type of technology in 3D printing. Figure 9 illustrates different materials in liquid and solid phases with the respective suitable and known AM technologies they can be used for, and Figure 10 displays a comparison between different AM technologies according to their specifications, such as printing speed, required energy and resolution.

Heat-affected zones due to rapid heating in the final production of the materials call for improvement in cracking susceptibility during deposition by producing equiaxed grain sizes. In this respect, fine grains yield enhances mechanical properties and yields better performance in handling crack propagation. Heat treatment for metals, ceramics and polymers should be considered, and it might be required for meeting final product quality standards.

### 2.6. Nature-Inspired Structures: Triply Periodical Minimal Surfaces (TPMS)

Controlling gas emissions of the power, automotive transportation and material fabrication industries requires employing catalysts with different, often complex characteristics. This can be achieved either by using a substrate (support) structure loaded with the active materials such as catalysts [155,156,157], or by fabrication of a geometrically complex catalyst or adsorbent. 3D printing and AM have offered the opportunity to design a substrate providing high surface area and high density for pass-through flow of gases or liquids [158,159]. Until recently, most adopted catalyst substrates were structured like honeycomb [160]. The unceasing efforts of enhancing the design of catalyst substrates allowed researchers to come up with novel 3D structures made from ceramics. These are now widely used in catalysis and adsorption applications, as they are able to decrease the pressure drop and enhance heat transfer effects and flow efficiency.

The term triply periodical minimal surfaces (TPMS) stands for 3-dimensional structures repeated in three different directions, x, y and z, with minimal surface, i.e., defined as surfaces with zero mean curvature [161]. TPMS structures are mathematically created as shown in Table 1 [63], and do not intersect in 3D space. TPMS structures can be naturally found in organic forms, or they can be synthetically created under certain conditions. In engineering, analogous material composites are being extensively investigated and reviewed in the literature owing to their remarkable properties and multifunctionality that play imperative roles in various applications.

TPMS structures have been investigated in various fields. Abueidda et al. [162] introduced a new type of 3D interpenetrating phase composite (IPC). They applied a finite element method (FEM) to predict multifunctional properties of these structures, showing that TPMS can be used as solid shell reinforcements in IPC. In addition, metal-ceramic IPC structures have been studied for different applications, such as braking materials, showing highly efficient friction and wear behavior similar to cast iron but with half of its density, thereby exhibiting potential in light-weight structures [163,164,165]. Their resistance towards thermal fatigue has enabled use in variable temperature applications according to del Rio et al. [166], and IPC structures proved to sustain their hardness in a wide range of applications with good mechanical integrity.

**Figure 11:**
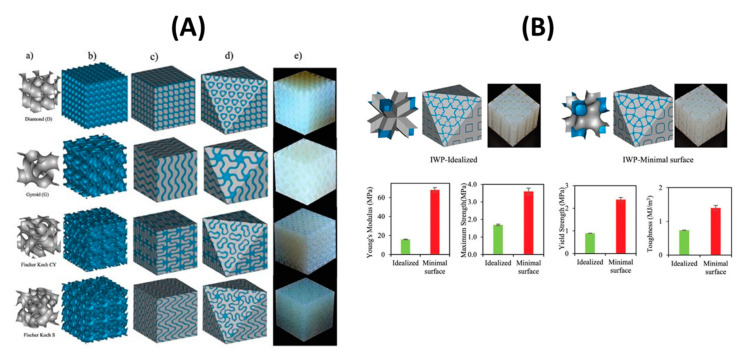
(**A**) Different TPMS topologies used as solid sheet reinforcement phases in IPC: (a) a single unit cell, (b) a 3 × 3 × 3 patterning of the TPMS, (c) the designed IPC, (d) a (111) plan cut to reveal the interconnectivity of the TPMS and (e) a sample fabricated using 3D printing. (**B**) TPMS-IWP vs. the idealized IWP. Reproduced with permission from [167]. Copyright Wiley-VCH, 2016.

Al-Ketan O. et al. [167] investigated a new type of 3D structure by reinforcing a soft matrix with hard material, resulting in the TPMS structure shown in Figure 11A. The volume fraction, defined as the volume of the material to void, has been varied using mathematical CAD software. For that purpose and as an example, the multi-material Connex 260 3D printer (Objet Geometrics, Billerica, MA, USA), which allows concurrent printing of two dissimilar materials with micrometer resolution, was employed to fabricate the IPC samples. The results show that TPMS Schoen’s I-graph and wrapped package graph (IWP) (Table 1) outperforms the idealized IWP, as shown in Figure 11B.

**Figure 12:**
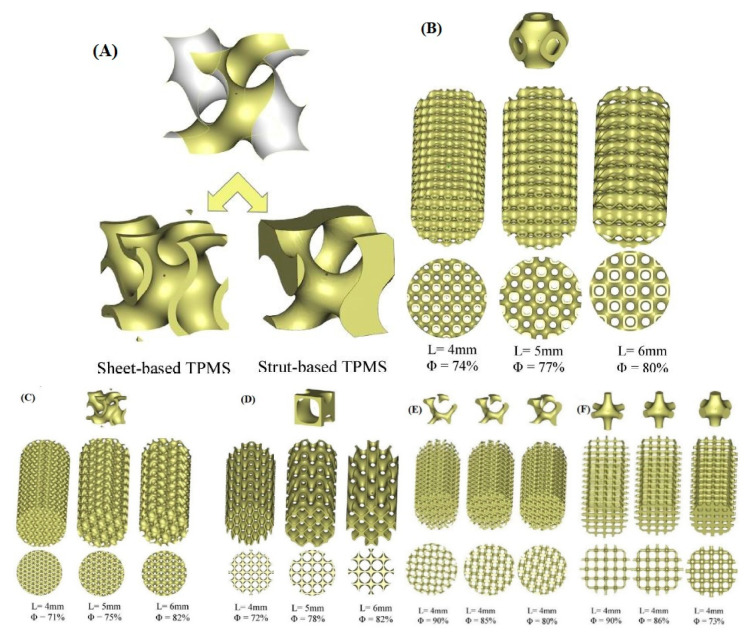
(**A**) Illustration of sheet-based TPMS and strut-based TPMS structures with different unit cell sizes (L) and macroporosities (Φ). (**B**) Primitive sheets, (**C**) gyroid sheets, (**D**) crossed layers of parallel (CLP) sheets, (**E**) gyroid struts and (**F**) primitive struts. Reproduced with permission from [168]. Copyright The American Ceramic Society, 2019.

The substrates used to support catalysts must undergo mechanical testing to evaluate their ability to withstand and function well under realistic conditions. Al Ketan, O. et al. [168] designed new structures based on the TPMS concept, whereby instead of periodic struts, a single, continuous, smooth and periodic surface sheet was employed. The 3D design modeled two different topologies, the strut-based and the sheet-based TPMS, as shown in Figure 12A. Their study demonstrated that the strut-based substrates exhibit stress localization leading to faster failure than the sheet-based substrates where the stress was found to be distributed homogeneously. A compilation of three designs of TPMS structures were fabricated and tested, i.e., the primitive, crossed layers of parallel (CLP), and gyroid structures, as shown in Figure 12B; their mathematical representations are listed below:

(3) CLP=sin z∗sin y−0.4(sin x∗cos z∗cos y)

(4) Gyroid=sin x×cos y+sin y×cos z+sin z×cos x

(5) Primitive=30×(cos x+cos y+cos z) +0.1×(cos x×cos y+cos y×cos z+cos z×cos x)+5

The TPMS structures were tested for pressure drop using a high temperature pressure drop (HTPD) apparatus under ambient temperature (298 K) and atmospheric pressure (98 kPa). The experiments were performed by setting three air flow velocities corresponding to the following Reynolds numbers: 1111 (0.9 m/s), 2221 (1.8 m/s) and 3332 (2.7 m/s).

**Figure 13:**
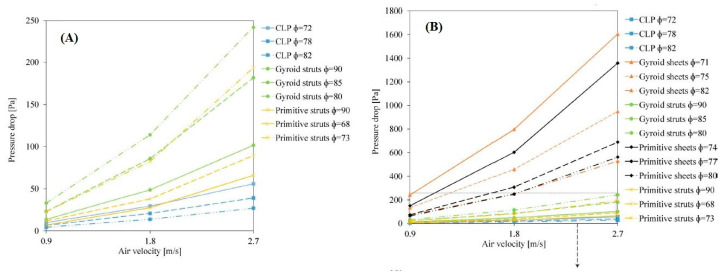
Pressure drop test according to the experiment design for (**A**) sheet-based substrates and (**B**) strut-based substrates for primitive, gyroid and CLP Structures. Reproduced with permission from [168]. Copyright The American Ceramic Society, 2019.

As can be seen in Figure 13, the gyroid sheets and primitive sheets exhibited the highest pressure, drop while the CLP showed the lowest pressure drop for both sheet- and strut-based substrates. It should be mentioned that all tested TPMS structures had similar ranges of porosity. Since the CLP geometry allows for the air to flow around minimum obstacles, unlike the gyroid and primitive ones, it exhibits the lowest pressure drop.

On the other hand, strut-based substrates exhibit lower pressure drops than sheet-based substrates. Although the struts have much higher porosities, this behavior can be explained by their geometric complexity. Results verified that CLP substrates exhibit a desirable behavior combining low pressure drop and good mechanical properties.

This work showed the efficiency of using AM in improving the design and thermal properties of catalytic supports using mathematically modeled structures optimizing material characteristics, minimizing pressure drop and enhancing performance.

## 3. Applications of 3D-Printing in Catalysis and Separations

### 3.1. 3D Printed Adsorbents for CO_2_ Capturing

3D printing has gained attention in fabricating mathematically modeled structures (Table 1) to produce shapes that cannot be produced using conventional methods, such as TPMS [52]. TPMS structures are gaining significant importance because of their unique functions obtained by maximizing the surface area of the structure and eliminating sharp edges, thereby offering outstanding mechanical properties by reducing stress concentrations. Recently, successful attempts in utilizing AM for separation processes were reported. Researchers have investigated the use of different materials in 3D printed monoliths, such as MOFs, zeolites, ceramics, etc., for CO_2_ capture with ambient air and exhaust gases, and found that they show potential advantages vs. pellets, such as higher mechanical endurance strength and lower pressure drops [169].

**Figure 14:**
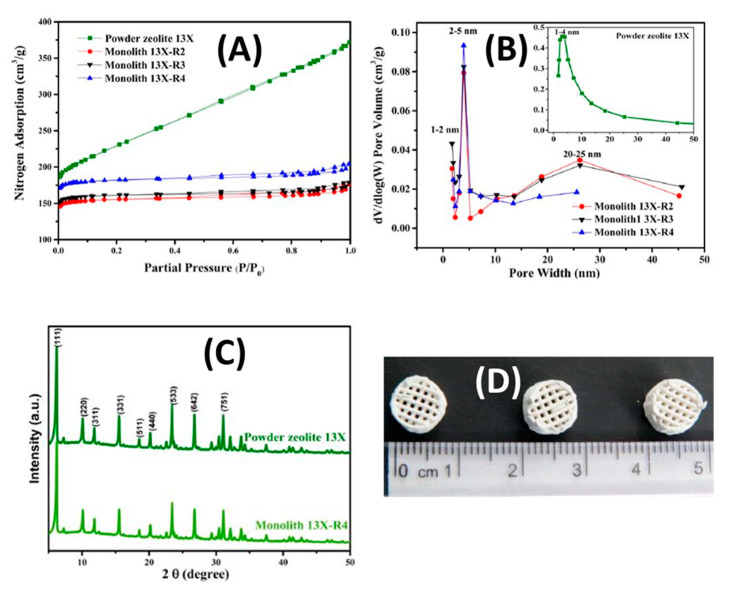
(**A**,**B**) N_2_ physisorption isotherms for 13X, pore size distribution of monoliths and powder zeolites. The pore size distribution was derived from the density functional theory (DFT) method, using the desorption branch of the N_2_ isotherm [65]. (**C**) XRD pattern for monolith R4 which includes 90 wt.% zeolites and powder analogue. (**D**) 3D printed monoliths from adsorbent material using robocasting technique. Reproduced with permission from [65]. Copyright American Chemical Society, 2016.

AM technology, particularly the robocasting technique, has been utilized by Harshul Thakkar et al. [65], to make 3D printing adsorbents monoliths for CO_2_ capturing from spacecraft, where attrition of adsorbent particles may pose serious health risks to astronauts. The 3D printer used in their research has high productivity, is expensive compared to other printers and resulted in printed monoliths with high catalytic efficacy and good recyclability. The self-standing struts, as shown in Figure 14D, are initially prepared from zeolite 13X, followed by the addition of plasticizers and binders in the paste used in the 3D robocasting printer.

To determine the physical properties of 3D printed monoliths, N_2_ physisorption and pore size distribution tests were used to compare them with their powder analogue. As illustrated in Figure 14A,B, the initial uptake takes place in the monolith at low relative pressures (0.0–0.05), corresponding to low pore size distribution (microporous). Relatively, CO_2_ uptake at high relative pressures is indicative of capillary condensation happening in mesopores, resulting in showing that the 3D printed zeolite monoliths follow isotherm IV type, while the powder analogue follows type I characteristic of microporous. At the same time, the BET surface areas of 13X monoliths were found to be 635 m^2^/g, whereas the micropore volumes (at P/P_0_ = 0.99) were calculated to be 0.24 and 0.25 cm^3^/g, respectively. All of these values were relatively lower than those for powder forms, as expected, as a result of lower zeolite content.

XRD was used to structurally compare the patterns of the monoliths with those of their powder analogues. The result shows that good crystallinity of the zeolites was retained, while some differences in intensity can be explained by the presence of plasticizers and co-binders. The peaks at 2θ = 6.2°, 15.6° and 30.9° correspond to the (111), (331) and (715) planes in the Faujasite (FAU) framework as shown in Figure 14C.

To improve mass transfer as one of the ultimate benefits of 3D printed structures, the plasticizer (clay and bentonite) needs to be removed via a calcination process. For the calcined monolith, thermal gravimetric analysis (TGA) showed a 10 wt.% total weight loss in the 200–700 °C range. The weight losses at <200 °C correspond to moisture desorption. For uncalcined monoliths, the weight losses appearing between 200 and 700 °C are associated with the decomposition of organic additives; whereas for calcined samples, small weight losses could be attributed to the loss of organic compounds trapped in the pore network during the sintering process and retained in the structure after calcination [114].

**Figure 15:**
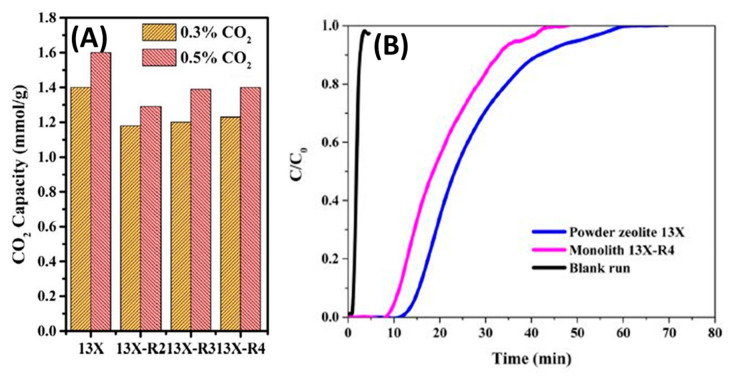
(**A**) CO_2_ adsorption capacities for 3D-printed monoliths and zeolite powders obtained at 25 °C using 0.3% and 0.5% CO_2_ in N_2_. (**B**) Breakthrough curves for 13X-R4 and 3D-printed monoliths and zeolite powders obtained at 25 °C and 1 bar. Reproduced with permission from [65]. Copyright American Chemical Society, 2016.

The dynamic adsorption performances of zeolite monoliths and powders were evaluated at 25 °C and atmospheric pressure, using a feed gas containing 0.5% CO_2_ in N_2_ at a flow rate of 60 mL/min; the corresponding CO_2_ breakthrough profiles are presented in Figure 15A. For 13X, the zeolite powders retained CO_2_ longer and exhibited longer breakthrough times than their monolithic counterparts, as shown in Figure 15B. Here the breakthrough width is defined as the difference between the time to reach 5% and 95% of the final composition [33]. Table 2 tabulates the breakthrough times for the samples.

In particular, for 0.5% CO_2_/N_2_, the 13X-R4 monolith showed a CO_2_ uptake of 1.39 mmol/g, which is 87% of that of 13X zeolite in the powder form. Moreover, as can be seen from these results, increasing the zeolite/binder weight ratio resulted in an increased CO_2_ adsorption capacity.

In another study, Thakkar et al. [62] utilized the robocasting 3D printing technique to obtain structured monoliths for CO_2_ capture and various gas separation applications. The adsorbent material was firstly synthesized in the lab, using a metal–organic framework, namely, MOF-74(Ni) and UTSA-16(Co), as a base material in addition to plasticizers and binders to control the shear thinning as a pre-step before deploying the slurry in the 3D printer. Finally, the monoliths were designed using CAD software for 3D printing.

**Figure 16:**
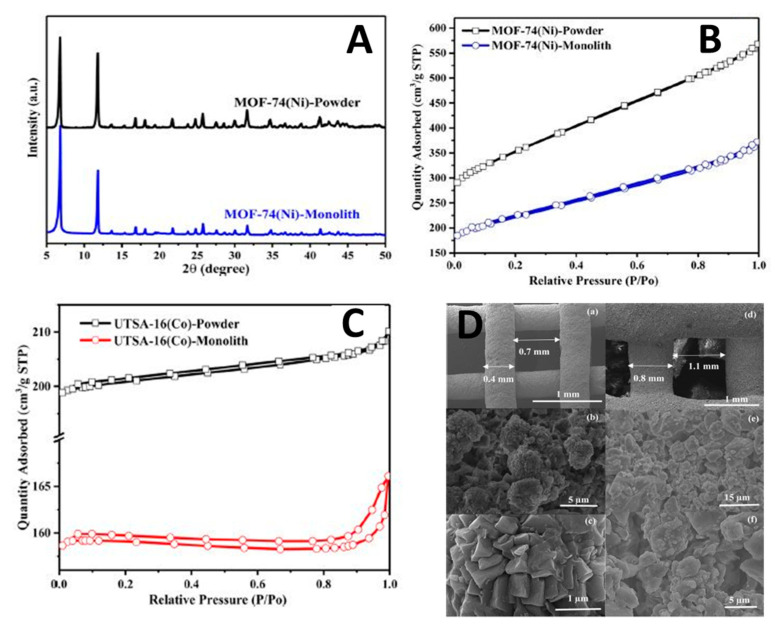
XRD patterns for 3D-printed (**A**) MOF-74(Ni) and UTSA-16(Co) monoliths with their powder counterparts. Nitrogen physisorption isotherms and pore size distribution curves for 3D-printed MOF monoliths: (**B**) MOF-74(Ni) and (**C**) UTSA-16(Co) and their corresponding powders. (**D**) SEM images of 3D-printed (a−c) MOF-74(Ni) and (d–f) UTSA-16(Co) monoliths. Reproduced with permission from [62]. Copyright American Chemical Society, 2017.

XRD studies verified that the crystallinity of the MOF has not changed during the printing process (Figure 16A), whereas N_2_ adsorption at 77K for both the MOF monolith and the powder showed a type I isotherm, indicative of microporous nature of the adsorption material, with the uptake of the monolith being much smaller than the powder.

Figure 16D shows the SEM imaging of the 3D printed samples. The results confirm the uniform pore structure of the 3D-printed MOF-74(Ni) monolith, similar to the MOF powder, with the pores having sizes in the range of 1−4 μm, but with smaller pore volume. For the 3D-printed UTSA-16(Co) monolith, a hysteresis loop appeared in the N_2_ isotherms shown in Figure 16C. Similarly to the MOF powder form, some mesopores were formed in the MOF structure during printing. This could be attributed to the effect of water added while preparing the paste. Additionally, it was reported that the additives used to control shear thinning for printing purposes led to sizable reduction of the surface area for MOF-74(Ni) up to 38% (from 1180 to 737 m^2^/g) and for UTSA-16 (Co) up to 30% (from 631 to 444 m^2^/g), as shown in Figure 16B.

Figure 16D shows the cross-sectional area with wall thicknesses and channel width of 0.4 and 0.7 mm, for the MOF-74(Ni) monolith, and 0.8 and 1.1 mm, for the UTSA-16(Co) monolith, respectively. The reason for printing monoliths with different sizes was that the viscosities of the pastes were different for the two MOFs; for UTSA-16(Co) with higher viscosity (1659 kg/m^3^), a less viscous paste was prepared to avoid blockage and enhance extrusion. This led to expansion while depositing layers, which eventually resulted in a larger wall thickness and channel width for this MOF than for MOF-74(Ni) with lower density (909 kg/m^3^).

The weight loss in the 310–350 °C range for 3D-printed MOF monoliths is associated with the PVA decomposition and accounted for 5% which is close to the nominal amount of PVA in the printed monoliths. In addition, peaks at 450–470 and 380–390 °C were found for the thermal decompositions of the MOF- 74(Ni) and UTSA-16(Co) structures, respectively. At temperatures above 600 °C, the differences in mass between the powders and 3D-printed monoliths were found to be ≈13% and ≈8% in MOF-74(Ni) and UTSA-16(Co), respectively, which were close to the nominal weight fractions of the binder used in the preparation step.

**Figure 17:**
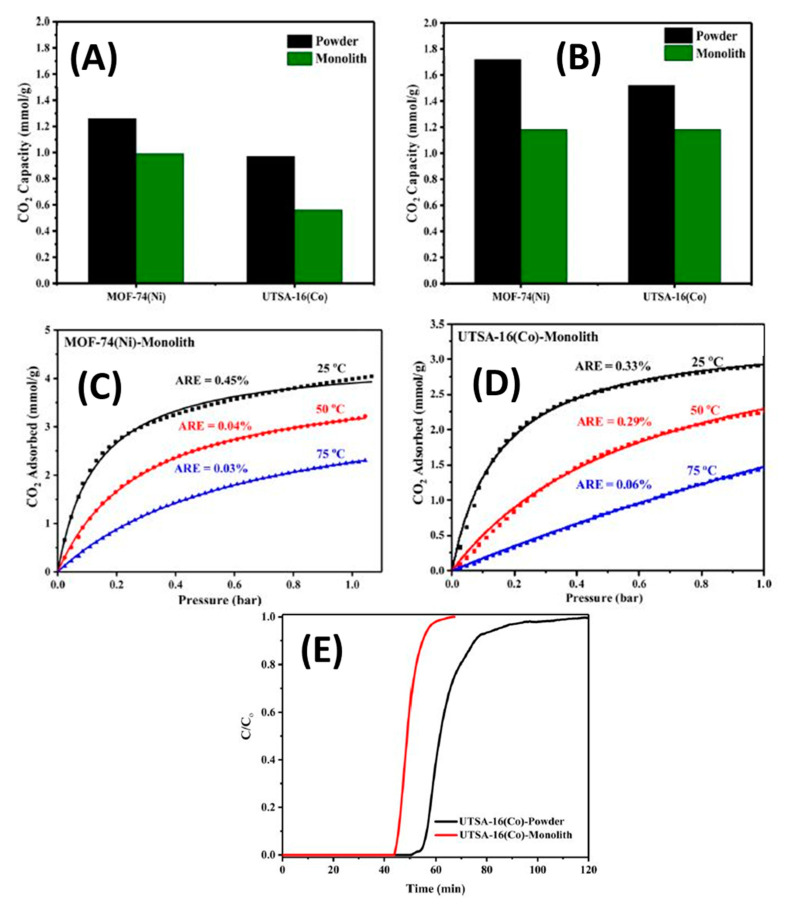
(**A**,**B**) CO_2_ capacities of 3D-printed MOF monoliths and corresponding powders under (A) 3000 and (B) 5000 ppm CO_2_/N_2_ at 25 °C and 1 bar [62]. (**C**,**D**) CO_2_ adsorption isotherms of 3D-printed (C) MOF-74(Ni) and (D) UTSA-16(Co) monoliths at 25, 50, and 75 °C. Symbols show the experimental data, and solid lines represent the fitted isotherms with the average relative error (ARE). (**E**) Breakthrough profiles of the 3D-printed UTSA-16(Co) monolith and its corresponding powder using 5000 ppm CO_2_/N_2_ at 25 °C. Reproduced with permission from [62]. Copyright American Chemical Society, 2017.

The CO_2_ capacities for both monoliths and their powder forms are shown in Figure 17. The CO_2_ uptakes values of 3D-printed MOF-74(Ni) and UTSA-16(Co) monoliths were found to be 1.35 and 1.31 mmol/g, respectively, at 0.5% CO_2_/N_2_—about 79% and 87% of those of MOF powders. The change in porosity of the 3D-printed UTSA-16(Co) monolith did not influence its capture capacity. In addition, the adsorption isotherms decreased by increasing the temperature. The samples were tested for adsorption at three different temperatures 25, 50 and 75 °C.

Lawson et al. [170] and coworkers who had previously investigated 3D printed zeolite monoliths for CO_2_ adsorption had also examined 3D printed MOFs using polymers to address their rheological limitations. Two MOFs were examined in their study: MOF-74 and HKUST-1. The results of CO_2_ adsorption capacities at 25 °C and 1 bar were 0.5 and 2.5 mmol/g for MOF-74 and HKUST-1 respectively. Using fractional uptake measurements to test the dynamic performances of the printed monoliths showed an increase in the diffusional resistances from 40% to 60% for the HKUST-1, while MOF-74 showed the steepest drop fractional uptake. It was found that a secondary growth for MOF-74 was necessary due to the decomposition of its crystals in order to restore the crystallinity of the MOF.

Torlon-MOFs were synthesized and prepared, whereas the HKUST-1 and MOF-74 were suspended in a polymer dope, and then printed directly through the robocasting technique. The HKUST-1 did not decompose like MOF-74, permitting it to be directly used in 3D printing with high loads. Meanwhile, the decomposed MOF-74 particles were used as seeds in secondary growth with lower loads.

Results showed that the HKUST-1 viscosity is much higher than is suitable for the 3D printer to operate, requiring an extremely low deposition speed and high extrusion pressure. In contrast, MOF-74 showed very low viscosity and shear thinning, which was attributed to the partial decomposition of the MOF.

Wang S. et al. [171] constructed mechanically robust binder-free zeolite monoliths (ZM-BF) with hierarchical structures for CO_2_ capture. Initially, halloysite nanotubes were used as an ink additive in addition to the precursor to reinforcement. The final 3D printed monolith showed promising results, with the compressive strength achieved of (5.24 MPa) being among the highest strengths of zeolite monoliths with binders. In addition, the CO_2_ uptake reached 5.58 mmol/g at room temperature and pressure, which is also the highest among all 3D printed zeolite monoliths. Interestingly, the binder-free zeolite monolith showed excellent performance in CO_2_ capture capacity at low partial pressures from natural gas, CH_4_ and flue gas, N_2_. In comparison to commercial adsorbents, the binder-free zeolite showed higher CO_2_ uptake, especially when compared to the commercial NaX powder form material. This can be due to the recrystallization process of zeolites with silica to alumina ratio lower than the commercial one [172,173].

Middelkoop et al. [174] reported 3D printed zeolite and carbon monoliths for natural gas sweetening and treating by physical adsorption of CO_2_ and H_2_S, i.e., the impurities existing in natural gas. The study compares the packed bed to monolith configurations for adsorbents. The results showed faster adsorption and desorption rates of monoliths than those for beads.

Zeolite 13X and carbon were utilized to build a matrix-like structure by direct writing technique to produce monoliths. The surface area was characterized using a BET instrument, and found for zeolite 13X beads to be 0.26 m^2^/g, which agrees with the literature [175,176]. The adsorption isotherms for zeolite beads follow type I according to IUPAC classifications, while the 3D printed monolith followed type I and II. The existence of type II was attributed to the presence of macropores. The average pore diameters of the 3D printed samples were found to be 2.75 and 3.5 nm for 13X and carbon respectively.

Lawson et al. [177] utilized additive manufacturing in producing 3D-printed monoliths with different wall porosity and cell density, to study the dynamic effect on diffusion rates. CO_2_ and N_2_ physisorption was performed under various conditions. The results showed a decreasing trend in CO_2_ mass transfer with increasing cell density, while a higher mass transfer rate was attributed to the microporous binder. Monoliths with different cell densities (200, 400, 600 cpsi) and porosities (0.23–0.46) were 3D printed. The results showed a surface area between 550 and 570 mm^2^/g, and all samples exhibited very high selectivity towards CO_2_ over N_2_. Three different strut thicknesses (cpsi-1, cpsi-2 and cpsi-3) for the same porous monolith were examined, showing no significant differences in adsorbing capacity.

Because the 200–600 cpsi monoliths were of low macro porosity, the adsorbate diffusion through the zeolite particles was limited by intra-particle diffusional resistance [178]. Thus, the lower the cell density, the better the performance in dynamic behavior, including mass transfer and diffusional rates. In order to overcome diffusional problems, researchers enhanced the macroporosity by increasing the concentration of the methylcellulose.

### 3.2. 3D Printed Catalysts for Reforming Reactions

#### 3.2.1. Metal/Ceramic 3-D Printed Catalysts

Nickel-based catalysts are used in the methane reforming reaction, which is employed for half of the world’s hydrogen production [179]. Due to the tubular nature of the catalyst there are several mass and heat transfer limitations, especially at the working temperature between 1100 and 1400 °C, reducing efficiency to the level of 65%. The novelty explored by current research is therefore to increase the efficiency and reliability of these catalysis reactions, especially because of their highly endothermic, heterogeneous and mass diffusion-limited nature. Subsequently, 3D printing must be able to address these catalyst fabrication limitations for these types of reactions that use more than one material in each catalytic system, and therefore have boundary layer limitations.

Reforming reactions [180] are vital in the petrochemistry industry thanks to their products, such as hydrogen and synthesis gas [8,10,26,27,181]. On the other hand, catalytic reforming can also be used to treat carbon dioxide emissions and reduce its effects when the dry reforming reaction is critical.

Tubío et al. [114] synthesized a woodpile structure with a Cu/Al_2_O_3_ catalytic system using a 3D printing technique, and then sintered at high temperature to enhance mechanical strength, and reach a high surface-to-volume ratio with controlled porosity of the produced copper-supported rigid structure. Their work focused on immobilizing the Cu on the Al_2_O_3_ in order to prevent the reaction from metal leaching. As a result, the produced device showed no leaching of Cu into the reaction medium, high catalytic efficiency and outstanding recyclability. Such 3D printed systems have become very important due to their ease in manufacturing, reactivity, recyclability and contamination resistance. Even though there is no catalytic application mentioned in this research work, it is of interest to include these major findings in this current review, as it highlights some aspects of structural/thermal stability of catalysts which are important to the reforming reaction conditions.

**Figure 18:**
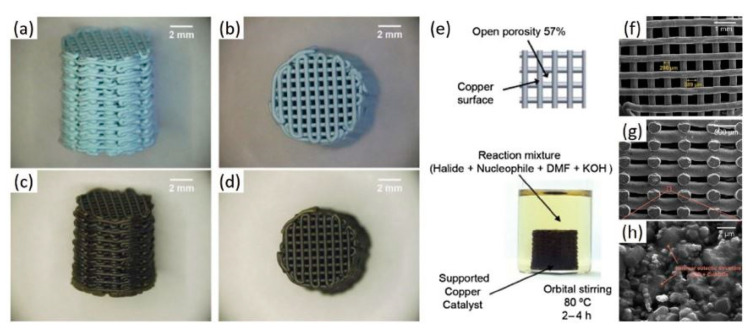
3D printed Cu/Al_2_O_3_ catalyst. (**a**,**b**) Optical images of the Cu/Al_2_O_3_ structure without sintering. (**c**,**d**) Optical images of the sintered Cu/Al_2_O_3_. (**e**) Schematic illustration and image of the experimental setup used for the catalytic tests. (**f**–**h**) SEM images of sintered woodpile structure fabricated from a concentrated Cu/Al_2_O_3_ ink deposited through a 410 μm nozzle. Reproduced with permission [114]. Reproduced with permission from [114]. Copyright Elsevier, 2015.

The 3D printed structure was made of Al_2_O_3_ base material with some heat treatment to make it stronger and more stable. As shown in Figure 18a–d, the layer pattern follows an arrangement of parallel rods separated by approximately 960 µm. The final 3D printed structure exhibited some shrinkage after drying, which led to minimizing of the rod spacing to 806 µm, while the rod diameter was measured to be 354 µm.

In order to ensure its reusability, the structure was heated to 1400 °C, i.e., the typical sintering temperature for alumina. While heat treating the structure, copper was immobilized and binders were removed. Finally, the woodpile structure showed no cracks despite a high shrinkage in diameter by about 29%, and open porosity of 57%. It was noticed that the color changed from blue to dark brown, which was explained by the transformation of Cu(NO_3_)_2_2.5H_2_O to CuO and CuAl_2_O_4_.

SEM imaging of the fabricated structure in Figure 18e–h indicates that sintering does not affect the morphology of the catalyst. Figure 18a–d also shows same interconnected rods with different colors and little deflection due to heat treatment. Figure 18f–h shows a dense assembly of Al_2_O_3_ crystals. A laminar structure of the eutectic phases can be observed clearly in this image, which indicates growth of the copper oxide phases (CuO, CuAl_2_O_4_) over the Al_2_O_3_ surface.

In summary, the ease of using 3D printing to fabricate such structures with high catalytic efficiency and outstanding recyclability, and no metal contamination, all make the additive technique a good alternative to also fabricating other metal/oxide heterogeneous catalytic systems.

Tianqing Zheng et al. [181], presented 3D printed porous structures made of FCC and BCC stainless steel crystal support structures for a Cu/Zn/Al/Zr catalyst loading to be used in SR. Stainless steel support structures are very well known due to their stable mechanical properties such as strength and toughness, enabling them later to be used in the methanol reforming process to produce hydrogen. The researchers focused on BCC and FCC structures with spherical shapes, due to their large surface area, which is beneficial for enhancing the reaction performance of the porous support.

**Figure 19:**
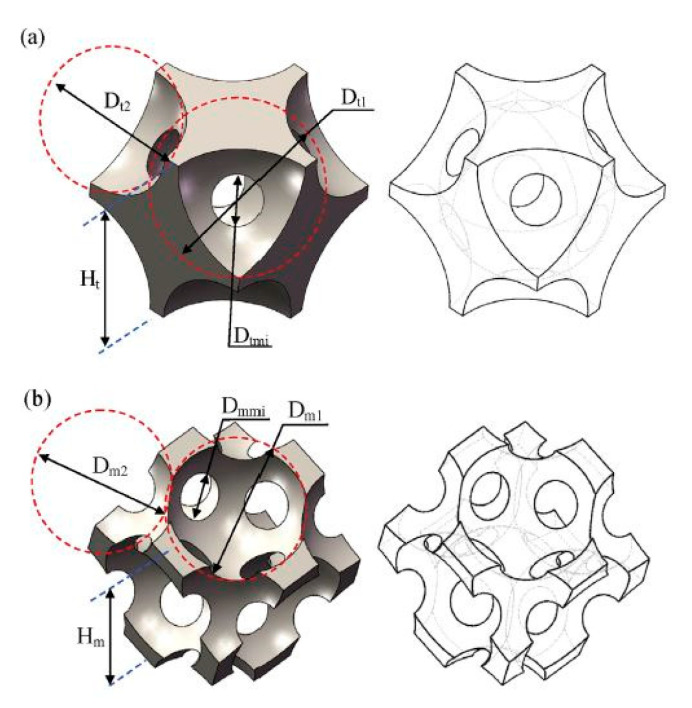
A cell of the designed porous structure with two different crystal structures, Body Centered Cubic (BCC) and Face Centered Cubic (FCC). For the porous cell of BCC H_t_ represents the edge length of the cube structure model, as shown in (**a**). Moreover, D_t1_ indicates the diameter of the atom at the center of the BCCS. D_t2_ represents the diameter of eight atoms at the apex angle. D_tmi_ indicates the diameter of the interconnected hole. Furthermore, for the porous cell of FCCs, H_m_ represents the edge length of the cube structure model, as shown in (**b**). D_m1_ indicates the diameter of atoms in the center of the FCCs. D_m2_ represents the diameter of eight atoms at the apex angle. D_mmi_ indicates the diameter of the interconnected hole [181]. Reproduced with permission from [181]. Copyright Elsevier, 2020.

Moreover, an example of a porous support made of a BCC crystal structure was designed via a Boolean operation, i.e., by removing the intersection between a fully built cube and the BCC structure model. Consequently, the support geometry was assembled by placing an array of the designed cell, as shown in Figure 19.

The researchers reported that copper was difficult to be 3D printed, due to its significant reflectivity to the laser beam (1 micron in wavelength), making it hard to absorb the laser energy. Consequently, stainless steel was used to build the porous support. Loading the catalyst material into the porous support was obtained through sequenced steps, starting by impregnating the catalyst precursor solution which contains Cu/Zn/Al/Zr into the support structure, and then drying it in the oven. Those two steps were repeated several times until the support was fully loaded with the catalyst material.

**Figure 20:**
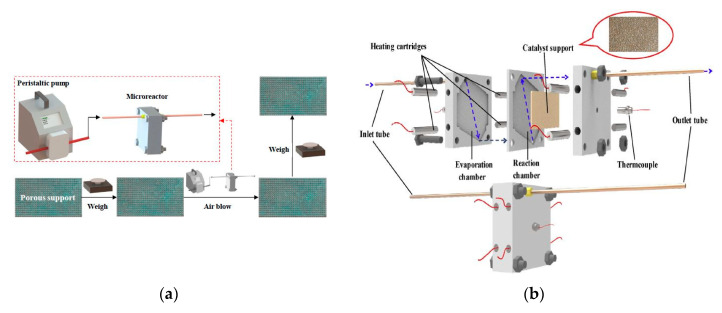
(**a**) Catalyst loading strength measurement starts by blowing dry air onto the loaded support and ends with weighing it to measure the weight difference. (**b**) Illustration of plate microreactor for methanol SR for hydrogen production. Reproduced with permission from [181]. Copyright Elsevier, 2020.

The catalyst loading strength was investigated by using an air pump directed into the porous support loaded with catalyst, to determine weight difference as shown in Figure 20a. The smaller mass difference indicates higher catalyst loading strength. In addition, testing the performance of the methanol SR microreactor with 3D printed and catalyst-loaded support is shown in Figure 20b. The methanol–water mixture was evaporated into gas in an evaporation chamber. Then, the gas was fed into the reaction chamber, and hydrogen production was monitored. The chemical reaction is expressed in Equation (6).

CH_3_OH + H_2_O ⇒ 3H_2_ + CO_2_; ∆H_298°_ = +49.4 KJ/mol (6)

It is noteworthy that the 3D printed support structure showed some deviations in dimensions compared to the 3D printed one. This led to certain differences in the surface area and thus the expected performance. The designed surface areas of BCC and FCC structures were 11.08 and 15.05 mm^2^/mm^3^ respectively; by measuring the surface area after manufacturing by Micro-CT they were found to be 6.47 and 7.69 mm^2^/mm^3^ respectively.

**Figure 21:**
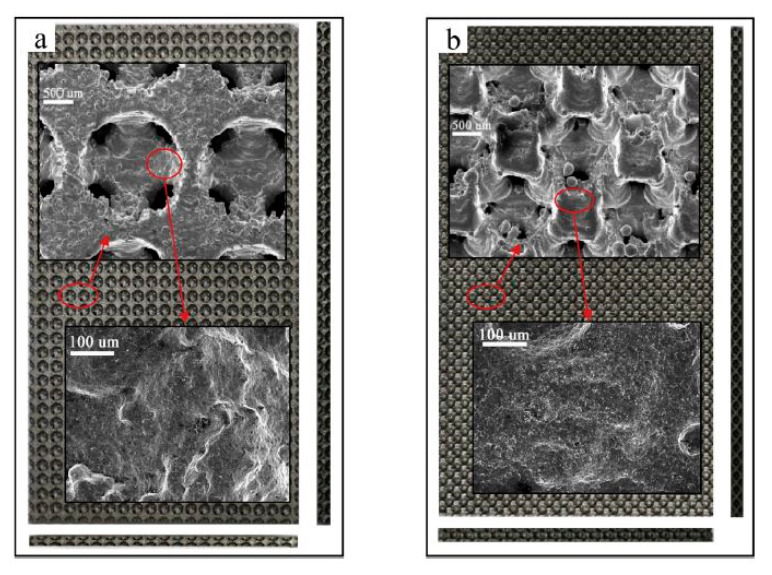
Optical image and SEM micrograph of 3D-printed porous stainless-steel supports with different crystal structures. (**a**) BCCs (**b**) FCCs. Reproduced with permission from [181]. Copyright Elsevier, 2020.

SEM imaging of the 3D printed porous support structure is shown in Figure 21 and compared to the CAD geometry for both crystal structures. As can be seen, 3D printing is capable of fabricating very small detailed porous structures, increasing its importance in the field of catalysis and separation.

In addition, energy dispersive X-ray analysis showed that the 3D printed porous support was loaded with the catalyst, confirming the presence of Cu/Zn/Al/Zr on both crystal structures. Moreover, the catalyst showed no difference in weight loss after exposing it for longer times, which indicates the strong binding of the catalyst to the stainless-steel support. This is attested through the reported time interval versus the loaded support mass for both BCC and FCC crystal structures.

**Figure 22:**
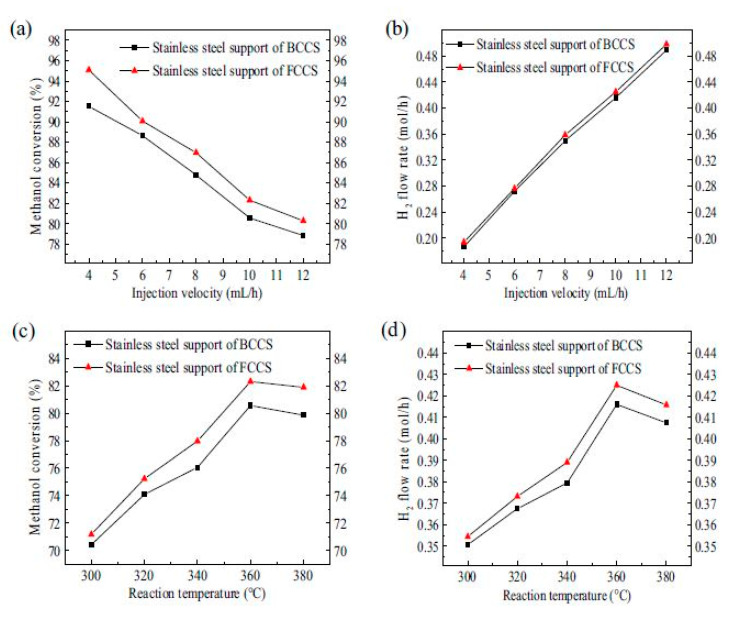
Reaction performances of microreactors with different 3D-printed porous stainless-steel supports for hydrogen production. (**a**) Methanol conversion. (**b**) H_2_ flow rate under different injection velocities. (**c**) Methanol conversion. (**d**) H_2_ flow rate under different reaction temperatures. Reproduced with permission from [181]. Copyright Elsevier, 2020.

Finally, the CO selectivity of the microreactor showed that the FCC 3D printed porous stainless-steel support structure has better selectivity than the BCC one. In addition, the methanol conversion and hydrogen flowrates were higher in the FCC crystal structure compared to the BCC by 3.9% and 3.8%, respectively, under same injection velocity. Moreover, BCC stainless steel supports had larger surface areas than FCC. Figure 22 illustrates the results; similar CO selectivity was obtained for different 3D-printed porous stainless-steel supports.

#### 3.2.2. Case Studies of 3D-Printed Hard Templates Used as Catalyst Matrices

Michorzyk et al. [66] used the 3D printer to build a template, which was then filled with the adsorption material for methanation reaction; and heated to burn the template, leaving behind a precise catalytic reactor. The study showed that the oxidative coupling of methane to the catalytic performance of monolithic catalysts at 820 °C for methane (MT1) had a 3.2% conversion of CH_4_; while for the aa Mn-Na_2_WO_4_/SiO_2_ system this was 26.7%, whereas the selectivity of CO_2_ was higher for MT1 at a percentage of 44.8%. Azuaje et al. [182] reported printing of an aluminum catalyst that acts as a Lewis acid in Biginelli and Hantzsch reactions.

Simge Danaci et al. [183] utilized robocasting 3-Dfiber deposition (3DFD) [184] to fabricate metal support structures and to coat it subsequently with Ni/Al_2_O_3_ as a reactor for methanation. The 3D printed support structure coated with Ni has been tested in a single tube over a variety of reported reaction conditions. The structured catalyst shows stability for 80 h in stream. The ordered 3D printed architectures played a role in the catalytic performance of alumina.

Michelle Kramer et al. [183] used additive manufacturing in producing a monolithic-structured support for catalyst substrate. They believe that 3D printing allows for tailoring the heat and mass transfer properties, overcoming the obstacle of conventional monolith structures having issues with heat transfer and natural convection of their walls. The research was focused on fabricating a reactor for steam-methane reforming. As a result of integrating the reactor wall into the catalyst substrate, the thermal conductivity was enhanced by 34% to reach 0.167 W/mk.

**Figure 23:**
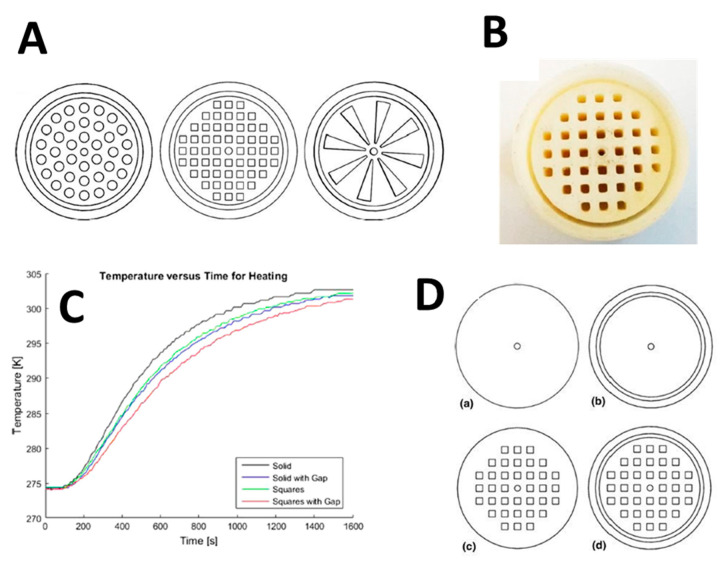
(**A**) Circular, square and biomimetic internal structure proposals. (**B**) Square-hole monolith with 35 mm diameter. (**C**) Heating of acrylonitrile butadiene styrene (ABS) catalyst substrates. (**D**) Different cross-sectional design for channels with substrate: (a) solid; (b) solid with gap; (c) squares; (d) squares with gap. Reproduced with permission from [119]. Copyright Nature, 2017.

The selective laser melting (SLM) technique was used to produce the support structure, as shown in Figure 23A. The cross-sectional view shows monolith structures and the gap between the reformer tube and catalyst support. The first concept was illustrated in Zamaniyan’s et al. [185] investigation, whereas the design shows a uniform pattern along its cross-section. The second design was investigated by Boger et al., and the last design was inspired by that of natural orange, with a structure allowing for efficient nutrient transfer throughout the fruit. The second sample shown in Figure 23B has a symmetric and clearly defined structure after being printed, and it was used for the experimental testing.

The properties of the final concepts of additive manufacturing substrates and their designs are illustrated in Figure 23D. It is clear that the solid substrate has the fastest response to heating, whereas the one with square gaps required a longer time for its response. This behavior can be explained by the lesser volume and lower amount of available material for heat transfer, which leads to lower heat conduction. In addition, heat conduction is known to be higher in turbulent environments in comparison to laminar flow, as a result of fluid mixing, as shown in Figure 23C.

Alongside the studies of coated support structures in multi-tubular or milli-channel reactor technology developed over the past decade, there have been recent advances in the development of all-in-one, multichannel catalytic reactors by direct 3D printing. New light is expected to be shed on finding ways to fully print active precursors distributed over a designed support structure, in comparison to the conventional way.

## 4. Conclusions

Compared to conventional manufacturing methods, additive manufacturing is becoming a powerful tool for aerospace, automotive, gas separation and catalysis applications. Different 3D printing techniques and technologies based on the printing scheme and material preparation process are discussed, along with their specific printing conditions and specifications. In addition, novel micro-structures based on mathematically generated geometries such as TPMS prove to offer promising alternatives. These can only be produced using additive manufacturing technologies, departing from conventional methods such as casting and forming. TPMS structures showed improved results in CO_2_ adsorption, since they allowed for better heat and mass transfer and low pressure drops. Thanks to their ability to be loaded with catalyst materials such as zeolites, MOFs and ZIFs, they became well-known in producing monolithic catalysts. For each new technique introduced, there are certain challenges to be addressed, such as metrology and related materials. Results showed that 3D printed structures play a main role in enhancing the uptake capacity of CO_2_ and excellent performance in reforming reactions. As comparatively little research has been performed in this area at the crossroads of mechanical and chemical engineering, various mathematically-based structures have been listed for future investigation.

## Figures and Tables

**Table 1:** Examples of triply periodical minimal surface (TPMS) unit cells [63].

Unit Cell Representation	TPMS Structure	Level Set Equation f(x,y,z)=C
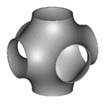	Schwarz Primitive	cosx∗cosy∗cosz−sinx∗siny∗sinz=C
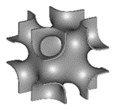	Schoen IWP	$\begin{array}{cc} 2(\cos x*\cos y+ & \cos y*\cos z+\cos x*\cos z)\\ & -\left(\cos2x+\cos2y+\cos2z\right)=C \end{array}$
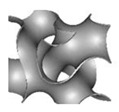	Schoen Gyroid	sinx∗cosy+siny∗cosz+sinz∗cosx=C
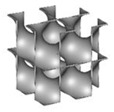	Schwarz Diamond	cosx∗cosy∗cosz−sinx∗siny∗sinz=C
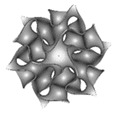	Fischer Koch S	$\begin{array}{cc} \cos2x*\sin y* & \cos z+\cos x*\cos2y*\sin z+\sin z\\ & \;*\sin x\cos y*\;\cos2z=C \end{array}$

**Table 2:** Breakthrough times for the samples [65].

Sample	t_5%_ (min)	t_50%_ (min)	t_95%_ (min)	Breakthrough Width (min)
**13X zeolite powder**	13	23	53	40
**13X-R4 monolith**	9	19	48	36
